# Health Tract, No. 14

**Published:** 1860-03

**Authors:** 


### HEALTH TRACT, No. 14.

### WINTER SHOES.

Like the gnarled oak that has withstood the storms and thunderbolts of centuries, man himself begins to die at the extremities. Keep the feet dry and warm, and we may snap our fingers in joyous triumph at disease and the doctors. Put on two pair of thick woollen stockings, but keep this to yourself; go to some honest son of St. Crispin, and have your measure taken for a stout pair of winter boots or shoes; shoes are better for ordinary, every-day use, as they allow the ready escape of the odors, while they strengthen the ankles, accustoming them to depend on themselves. A very slight accident is sufficient to cause a sprained ankle to a habitual boot-wearer. Besides, a shoe compresses less, and hence admits of a more vigorous circulation of blood. But wear boots when you ride or travel. Give directions also to have no cork or India rubber about the shoes, but to place between the layers of the soles, from out to out, a piece of stout hemp or tow-linen, which has been dipped in melted pitch. This is absolutely impervious to water—does not absorb a particle, while we know that cork does, and after a while becomes "soggy" and damp for a week. When you put them on for the first time, they will feel "as easy as an old shoe," and you may stand on damp places for hours with impunity.

### CURE FOR CORNS.

Corns are caused by too tight or too loose shoes, and sometimes in the bottoms of the feet by the wooden pegs protruding through the soles of the shoe, by the neglect of the maker to rasp them off sufficiently smooth.

Medical books record cases where the injudicious paring of corns has resulted in mortification and death. The safest, the best, the surest plan is to never allow a corn to be touched with any thing harder than the finger-nail. As soon as it becomes troublesome enough to attract attention, soak the foot fifteen minutes, night and morning, in quite warm water; then rub two or three drops of sweet oil into the top of the corn, with the end of the finger. Do this patiently for a couple of minutes. Then double a piece of soft buckskin, something larger round than a dime, rather oblong. Cut a hole through it large enough to receive the corn, and thus attach it to the toe. This prevents pressure on the corn, which always agravates it, and in less than a week the corn will generally fall out, or can be easily picked out with the finger-nail, and will not return for many weeks or months; and when it does return, repeat the process. No safer or more efficient plan of removal has ever been made known.

### NEW SHOES MADE EASY.

All part from an old shoe with special reluctance, because of the easiness of its adaptation to the foot. To put on a "bran new" boot or shoe, with the easy fitting of the discarded old one, is well worth knowing how to do. It is only necessary to keep a secret. Before you have your measure taken, put on two pair of thick stockings, and let Crispin go ahead. The new pair will be almost as easy as the old.

